# Frequency-Dependent Reduction of Cybersickness in Virtual Reality by Transcranial Oscillatory Stimulation of the Vestibular Cortex

**DOI:** 10.1007/s13311-023-01437-6

**Published:** 2023-09-18

**Authors:** Alberto Benelli, Francesco Neri, Alessandra Cinti, Patrizio Pasqualetti, Sara M. Romanella, Alessandro Giannotta, David De Monte, Marco Mandalà, Carmelo Smeralda, Domenico Prattichizzo, Emiliano Santarnecchi, Simone Rossi

**Affiliations:** 1 Siena Brain Investigation & Neuromodulation Lab (Si-BIN Lab), Unit of Neurology and Clinical Neurophysiology, Department of Medicine, Surgery and Neuroscience, University of Siena, Siena, Italy; 2 Oto-Neuro-Tech Conjoined Lab, Policlinico Le Scotte, University of Siena, Siena, Italy; 3 Health Statistics, University La Sapienza, Roma, Italy; 4 Precision Neuroscience & Neuromodulation Program, Gordon Center for Medical Imaging, Massachusetts General Hospital, Harvard Medical School, Boston, MA USA; 5 Otolaryngology, Department of Medicine, Surgery and Neuroscience, University of Siena, Siena, Italy; 6 Siena Robotics and Systems (SiRS) Lab, Department of Information Engineering and Mathematics, University of Siena, Siena, Italy

**Keywords:** Cybersickness, Virtual reality, Neuromodulation, Transcranial alternating current stimulation, Vestibular system

## Abstract

Virtual reality (VR) applications are pervasive of everyday life, as in working, medical, and entertainment scenarios. There is yet no solution to cybersickness (CS), a disabling vestibular syndrome with nausea, dizziness, and general discomfort that most of VR users undergo, which results from an integration mismatch among visual, proprioceptive, and vestibular information. In a double-blind, controlled trial, we propose an innovative treatment for CS, consisting of online oscillatory imperceptible neuromodulation with transcranial alternating current stimulation (tACS) at 10 Hz, biophysically modelled to reach the vestibular cortex bilaterally. tACS significantly reduced CS nausea in 37 healthy subjects during a VR rollercoaster experience. The effect was frequency-dependent and placebo-insensitive. Subjective benefits were paralleled by galvanic skin response modulation in 25 subjects, addressing neurovegetative activity. Besides confirming the role of transcranially delivered oscillations in physiologically tuning the vestibular system function (and dysfunction), results open a new way to facilitate the use of VR in different scenarios and possibly to help treating also other vestibular dysfunctions.

**Supplementary Information:**

The online version contains supplementary material available at 10.1007/s13311-023-01437-6.

## Introduction

Although virtual reality (VR) is, and in the near future will become even more, pervasive in our lives, most users experience a constellation of debilitating symptoms (nausea, dizziness, discomfort) known as cybersickness (CS). It is estimated that up to 95% of people wearing head-mounted VR displays experience some degree of these symptoms, which can be severe enough as to lead to abandonment of VR immersion in up to 15% of cases [1]. This problem has a serious and transversal impact on the many applications of VR, ranging from domestic first-person games and simulators to healthcare scenarios [2], including rehabilitation [3] and desensitisation psychotherapy strategies [4], training and performance during surgical interventions [5] and military applications: for example, the reproduction of virtual battlefield exercises and weapons production [6–8] or the pre-spaceflight training of astronauts immersed in special environments replicating the International Space Station [9, 10].

Similar to motion sickness (or kinetosis) syndrome [11], CS is thought to result from the continuous mismatch in the integration between vestibular, visual and proprioceptive inputs. However, in the case of CS, the primary mechanism responsible for the sensory mismatch (or conflict) [11] is thought to be “vection”, that is the illusion of self-motion without appropriate vestibular and proprioceptive feedback, rather than motion for kinetosis. Although mismatch is not the only theory behind the origin of CS [1], the sensory conflict gives rise to a dysfunction that primary involves the “vestibular network”, a widespread network (called the human vestibular network) that includes at least the autonomic, sensorimotor and cognitive domains [12].

The mismatch has its clear neurophysiological signatures in a widespread and progressive increase in low-frequency delta (1–2 Hz) electroencephalographic (EEG) oscillations [those that better activate the vestibular cortex during peripheral galvanic stimulation [13] in temporoparietal and occipital regions, as long as the symptomatology worsens [14, 15]. Such relationship between slow-wave EEG activity in the vestibular network and motion sickness has recently been demonstrated by inducing symptoms of motion sickness in healthy individuals not usually reporting them by transcranial alternating current stimulation (tACS) applied at 1 or 2 Hz, suggesting a robust causal relevance of slow-wave oscillatory activity of the vestibular cortical network to symptom generation [16].

Interventions using biophysically modelled tACS to reach the vestibular cortex in a rare vestibular areflexic patient with chronic symptoms of nausea, oscillopsia and postural instability, led to the observation that a higher stimulation frequency (i.e. 10 Hz) drastically reduced these symptoms [16]. We therefore reasoned that a similar approach could be used in healthy subjects to reduce CS during a standardised VR experience such as a rollercoaster ride. Mechanistically, a higher stimulation frequency might disrupt symptoms’ oscillatory processing at 1 or 2 Hz by phase interference [17], thereby reducing them. Alternatively, but not exclusively, entrainment of local oscillatory alpha activity, the suppression of which is a neurophysiological signature of vestibular activation [18], could disrupt the functioning of the vestibular regions and thereby also reducing symptoms. Following this line of reasoning, different tACS frequencies, such as alpha and delta applied to the vestibular cortex, may act, respectively, either as frequency-dependent “healing” currents or as inducers of motion sickness itself [16]. We also used a peripheral index of neurovegetative activity to demonstrate online behavioural CS reduction, such as the galvanic skin response (GSR), which reflects eccrine sweat gland activity [19] and is related to vestibular system (hyper)function [20, 21] that drives motion sickness symptoms [22, 23].

## Materials and Methods

### Participants

Forty-one healthy young adults (all right-handed; 25 males and 16 females; mean age: 26.5 ± 3.1 years; education: 15 ± 2.8 years) were recruited from undergraduate medical students at Siena Medical School and postgraduate students at the Santa Maria alle Scotte University Hospital of Siena, Italy. Four of them withdrew for personal reasons; therefore, analyses were performed on 37 subjects (25 males and 12 females; mean age: 26.3 ± 2.8 years; education: 15 ± 2.8 years).

The sample size was determined on the following basis: based on preliminary measures of the duration (in seconds) of self-reported CS nausea (i.e. the main outcome measure) in a control condition (no stimulation), the statistical distribution appeared to be approximately log-normal with a mean of 40 s and a standard deviation of the same magnitude. Such a skewed distribution is typical in biological experiments and requires a log transformation to improve the Gaussianity and control for possible outliers. By applying formulas proposed by Armitage [24], mean and SD of log-transformed data in the sham condition are expected to be similar to control condition and thus ln(40) = 3.69 log(s) with a $\mathrm{var}\left(\mathrm{y}\right)\cong {\left(\frac{\mathrm{dy}}{\mathrm{dx}}\right)}^{2}\frac{1}{\mathrm{X}\,=\,\mathrm{E}\left(\mathrm{x}\right)}\mathrm{ x\ var }\ (\mathrm{x})$ and, since y = ln(x), $\mathrm{var}\left(\mathrm{ln}\left(\mathrm{lenght}\right)\right)={\left(\frac{1}{40}\right)}^{2}\mathrm{x}{40}^{2 }=1$ and thus SD = 1 log(s). We considered a reduction of 20 s (expected for 10 Hz-tACS) as the minimum “clinically relevant” difference and an increase of 20 s (expected for 2 Hz-tACS, on the basis of a previous study [16]) as a secondary outcome. On a log scale, these effects correspond to log(20) = 2.99 and log(60) = 4.09, respectively (SDs can be assumed homogeneous and equal to 1 log(s)). Since we were interested in two specific contrasts (10 Hz-tACS vs. sham and 2 Hz-tACS vs. sham), alpha was set at 0.05/2 = 0.025. Power was set at 0.80. As this was a within-subjects study, we also assumed that the pairwise correlation between the measures in the three experimental conditions was around 0.7. G*Power 3.1 [25] indicated that in order to have a 80% probability of detecting a statistically significant (with a two-sided alpha 0.025) a decrease from 3.69 log s to 2.99 log s (corresponding to a change in CS length from 40 to 20 s), 15 subjects are required and to have a 80% power of detect as statistically significant (with a two-sided alpha 0.025) an increase from 3.69 log s to 4.09 log s (corresponding to a change of CS length from 40 to 60 s), 39 subjects are required. Allowing the possibility of some drop-outs, we recruited 41 subjects.

Subjects with a history of epilepsy, sleep disorders, migraine, psychiatric medication and history of other neurological or psychiatric disorders were excluded. Subjects were screened for their susceptibility to kinetosis using the Motion Sickness Susceptibility Questionnaire-Short form (MSSQ-Short) (score cut off 12) [26]. Each subject agreed to participate in the study and signed a written informed consent; the research was approved by the Local Ethics Committee (Code: Brainsight 21–24).

### Experimental Design

**Fig. 1:**
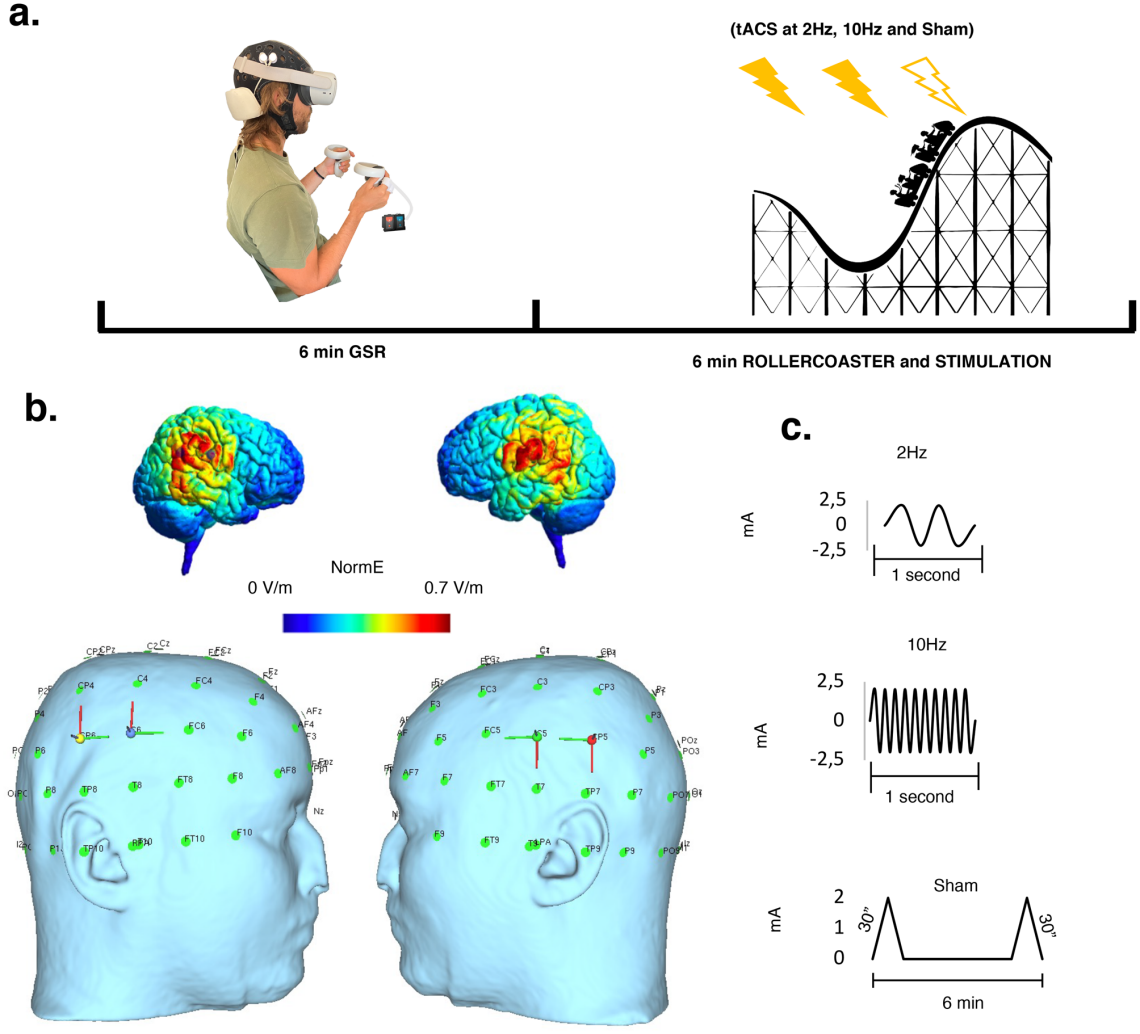
Methodology. **a** The subject immersed in the rollercoaster ride wearing the VR headset and GSR electrodes. The same set up was used in all the three stimulation conditions (each one lasting 6 min); in the 6 min preceding the VR experience, basal GSR activity was recorded. **b** tACS montage with 4 electrodes was chosen to stimulate the PIVC and PIC bilaterally: C5 (1.15 mA), C6 (−1.15 mA), CP5 (1.35 mA), and CP6 (−1.35 mA). The figure graphically shows the arising E-field (represented in NormE) resulting from the montage in V/m on a healthy example subject. **c** Sinusoids showing the three stimulation conditions (2 Hz, 10 Hz, sham) that were applied randomly

Figure 1 shows the experimental setting and design. Subjects were tested under the same experimental protocol, in the middle hours of the day, within a double-blind study design. Blindness was implemented using the MATLAB programming language, which randomly assigned a number to each condition without the experimenter’s knowledge. Also using the same script, conditions were randomised equally across participants. All subjects were naïve to tACS and unaware of the aims of the study. Therefore, even if they experienced some different sensation, they would not be able to attribute it to a particular tACS condition.

The experiment was conducted in a quiet environment to minimise the influence of the external stimuli. The protocol included four sequential trials during an Internet-available VR rollercoaster ride experience (epic rollercoaster) displayed through a head-mounted Oculus Quest 2. Each trial lasted 6 min and was 15 min apart from the next: the first trial consisted of a training session without any stimulation; the following three trials, whose order was randomised, included active tACS at different frequencies (2 Hz, 10 Hz) and sham (placebo). Stimulation at 10 Hz represented the experimental condition, while 2 Hz represented the frequencies that better activated the vestibular cortex during galvanic peripheral stimulation in neuroimaging studies [13] and induced motion sickness when applied to the vestibular cortex [16], while sham stimulation represented the control condition.

We preferred to perform the whole experiment in a single day in order to avoid the bias of possible habituation to CS with repeated exposures to VR in consecutive days [27], an effect that is not reported when VR exposures are separated by less than 1 h. The risk of a possible CS accumulation effect was minimised by randomising the conditions, while the risk of a carry-over effect of tACS sessions was excluded a posteriori by including this factor in the linear mixed model (see the “Results” section).

Participants engaged in the VR game were seated in a chair. Subjects were asked to verbally report the beginning and end of the periods of discomfort they experienced. The experimenter, blind to the type of stimulation, used a chronometer to record each period of reported discomfort during the different phases of the ride. The rollercoaster simulator (which is available online) allows for sudden changes in speed with rapid accelerations, rapid ups and downs interspersed with sections of straights. Players had to do nothing more than sit passively on the chair and report when they felt nausea or discomfort. At the end of the ride, they were asked when they would feel ready to take another ride (i.e. recovery time).

### Transcranial Alternating Current Stimulation (tACS)

High-definition tACS was delivered via a 32-channel hybrid EEG/tCS neurostimulation system (Starstim; Neuroelectrics, Barcelona, Spain). The device was wired by cable to the computer. Hybrid electrodes (NG Pistim) were used, consisting of an upper part containing the sintered Ag/AgCl core with a diameter of 12 mm, screwed to a lower base covering a circular area of approximately 3.14 cm^2^ which was covered. The electrodes were placed on a 32-channel neoprene EEG headset with holes corresponding to the positions of the International 10–20 EEG system. The scalp area below the electrode was prepared by inserting 15 ml of sterile sodium chloride solution (0.9%) to avoid discomfort on the skin and to reduce impedances, which were always kept below 20 kOhm. Gel (Signa, Parker Laboratories, Inc.) was applied to optimise signal conductivity and lower impedance. Electrode impedance was checked before starting each tACS session to ensure safety and maximum efficacy of stimulation, as well as to familiarise participants with the tACS-induced scalp sensations (e.g. tingling). tACS was applied at a maximum intensity of 2 mA on each electrode and a total of 4 mA across all electrodes, preceded by a 30-s ramp-up period and followed by a 30-s ramp-down period, while research and clinical staff were carefully monitored for any side effects throughout the duration of each session. For sham stimulation only ramp-up and ramp-down of 30-s was set, with no stimulation in between.

### Biophysical Modelling

To identify the correct electrode montage for our target we used an open source simulation software (SimNIBS v3.2). Through computational modelling with the Finite Element Method (FEM), SimNIBS integrates segmentation of magnetic resonance imaging (MRI) scans, mesh generation and E-field calculation to project current distribution and realistically calculate the electric field generated by different noninvasive brain stimulation (NIBS) techniques [28]. The software provides a realistic volume conductor head model, which is created by default in the FEM model generated using the T1-and T2-weighted images and segmentation from the SimNIBS example dataset [29]. The data sample was acquired from a healthy subject under the approval of the Ethics Committee of the Medical Faculty of the University of Tübingen [30]. The data correspond to a healthy subject (Ernie) and include white matter, grey matter, cerebrospinal fluid, bone and scalp tissue volumes. In our simulation, we kept the default isotropic conductivities [28] corresponding to grey matter: 0.276 S/m, cerebrospinal fluid: 1.790 S/m, bone: 0.010 S/m, scalp: 0.250 S/m [16]. The final mesh, including grey and white matter, scalp, bone and cerebrospinal fluid, comprises approximately 200,000 nodes and 3.6 million tetrahedral elements (see [30] for further modelling details).

Bilateral parieto-insular-vestibular cortex (PIVC), *x* =  − 43, *y* =  − 14, *z* = 17 (left) and *x* = 40, *y* =  − 14, *z* = 18 (right) and posterior insular cortex (PIC) *x* =  − 42, *y* =  − 36, *z* = 23(left) and *x* = 58, *y* =  − 34, *z* = 17 (right) [31] were considered as target areas. According to the simulation and the model, we identified a montage able to reach both target regions. Specifically, we placed 4 electrodes at the level of C5, CP5, C6 and CP6 (Fig. 1b). At the CP5 level, an intensity of 1.35 mA with a phase angle of 0° was used; at the CP6 level, an intensity of 1.35 mA with a phase angle of 180° was used; at the C5 level, an intensity of 1.15 mA with a phase angle of 0° was used; and at the C6 level, an intensity of 1.15 mA with a phase angle of 180° was used, for a total of 2.5 mA. Intensities inequalities were generated by the model [28].

### GSR Recording

In a subset of 25 subjects (14 males; 11 females) galvanic skin response was measured using the Neulog GSR logger sensor device (NUL-217) with two GSR probes attached by durable rubber-coated wires and two white Velcro finger connectors. As we were interested in tonic changes in GSR activity throughout the ride, the sensors were placed on the fingers [32]. Due to the head-mounted Oculus, sensors could not be placed on the forehead, the site most sensitive to phasic changes of skin conductance [11]. However, either phasic or tonic GSR changes are known to correlate with the severity of motion sickness [32]. Skin conductance activity was recorded before (6 min) and during (6 min) VR experience for each condition of stimulation separately. Measures of tonic GSR activity were expressed in microsiemens.

### Outcome Measures and Data Analysis

#### Cyber Sickness Nausea, Recovery Time and GSR

The primary aim was to verify the duration of self-reported CS nausea during the different phases of the rollercoaster ride (acceleration/deceleration, downhill, uphill, turns etc.) in the different stimulation conditions. Subjects were asked to verbally report each time a nausea sensation occurred and then disappeared. The length (in seconds) of these epochs was recorded and then summed up at the end of each condition. In addition to the epochs of discomfort during the VR experience, subjects were asked to report when these types of sensations ended (recovery time) after the session ended. GSR data refer to the difference between the GSR recorded during VR and before VR for each condition. A standardised side-effect questionnaire covering general discomfort, headache, itching and tingling during tACS [33] was also administered after each experimental condition.

### Statistical Analysis

Differences in CS nausea, recovery time, GSR and side effects were tested using IBM SPSS statistics 26 software. In behavioural data analysis, self-reported CS nausea and recovery time are the dependent variables, and the applied stimulation frequency is the independent variable. In physiological data analysis, GSR is the dependent variable, and stimulation frequency is the independent variable.

The Shapiro-Wilk test was used to test the normality of the data distribution. A non-normal pattern distribution was found for CS nausea reported during the 2 Hz-tACS (W = 0.783, *p* < 0.001), 10 Hz-tACS (W = 0.752, *p* < 0.001) and sham (W = 0.918, *p* = 0.008) conditions. The expected approximation to log-normal probability distribution was confirmed for 2 Hz-tACS and partially for 10 Hz-tACS, as indicated by the increase in the Shapiro-Wilk statistic after log-transformation (better fit to Gaussianity) in these two conditions; it should be noted that log-transformation did not improve the fit to Gassianity for the sham-tACS condition.

Linear mixed model (LMM) was used for CS length, recovery time, GSR and side effects. Compound symmetry was initially assumed, but the unstructured covariance matrix was also included to check the robustness of the results.

Correlation analyses were performed using a two-tailed Spearman’s test between MSSQ scores and CS duration, MSSQ and recovery time duration, GSR and CS length, GSR and recovery time length, CS nausea experienced during sham and effects of the 10 Hz-tACS.

Finally, linear regression with regression to the mean adjustment was used to test the predictivity of the level of CS nausea experienced during sham on the therapeutic effect of the 10 Hz-tACS. The significance level was set at 0.05 for each test. All graphs were generated with GraphPad Prism.

## Results

### Cyber Sickness Nausea

As the following results are obtained with a sample size based on certain assumptions and on an established effect size (see the “Materials and Methods” section), in order to interpret them correctly, we first checked the consistency between the assumptions and the observed outcomes. We assumed that the CS length in the sham condition was log(40) = 3.69 with a SD(log-scale) = 1. Quite differently, the observed CS length in the sham condition was 2.76 with a SD = 1.6. The other key assumption for the within-subjects design is the correlation between repeated measures, and we assumed a correlation of *r* = 0.7. Even this correlation was different than expected and was *r* = 0.59 between 2 Hz-tACS and sham and *r* = 0.47 between 10 Hz-tACS and sham. Thus, in the control condition (i.e. sham), we observed less nausea than expected (and with higher variability) and a lower within-subject correlation than expected. Together, these two deviations reduced the power of the study (e.g. with a sample size of 39 subjects, the power to detect as statistically significant (at alpha level 0.025) an increase in CS length from 15.8 (2.76 log s) to 35.8 s (3.58 log s) with a common SD = 1.6 (log s) is 0.74).

#### Whole Sample

According to the linear mixed model and assuming “compound symmetry”, the null hypothesis of no difference between the three conditions could be rejected (F(2,72) = 4.299, *p* = 0.017). The first planned comparison 10 Hz-tACS vs sham yielded a mean value of − 0.50 (95% CI: − 1.13, 0.13; *p* = 0.131) and the second planned comparison 2 Hz-tACS vs sham yielded a mean value of + 0.24 (95% CI: − 0.39, + 0.87; *p* = 0.725). The significance of the LMM is mainly due to the comparison between the two real stimulations: 10 Hz-tACS vs. 2 Hz-tACS resulted in a mean value of − 0.74 (95% CI: − 1.37, − 0.11; *p* = 0.016). As a sensitivity analysis, the “unstructured” covariance matrix was considered, and very close results were observed (F(2,36) = 4.313, *p* = 0.021). Thus, the primary outcome, 1 log-point decrease of CS with 10 Hz-tACS vs. sham, was not reached because (1) the assumptions were “optimistic” (higher CS and lower variance in the sham condition) and (2) the observed effect size was smaller than expected. The only significant difference occurred when the non-significant increase in CS observed with 2 Hz-tACS was compared with the non-significant decrease in CS observed with 10 Hz-tACS.

#### “Capable of Improvement” Sample

However, the discrepancy between the assumptions/hypotheses and the observations can be partially explained. Indeed, in order to reliably test whether 10 Hz-tACS was able to reduce CS, only subjects who experienced at least a time > 0 of CS during the sham should be considered; similarly, in order to test whether 2 Hz-tACS was able to increase CS, subjects who experienced the maximum CS value (150 s) during the sham should be excluded. We observed that 8 subjects had a CS length = 0 and none had a CS length = 150 during sham. After excluding them, we first verified a smaller discrepancy between assumptions and observations: the mean value of log(CS) in the sham condition was 3.52 (slightly lower than expected) with an SD = 0.83 (slightly lower than expected).

**Fig. 2:**
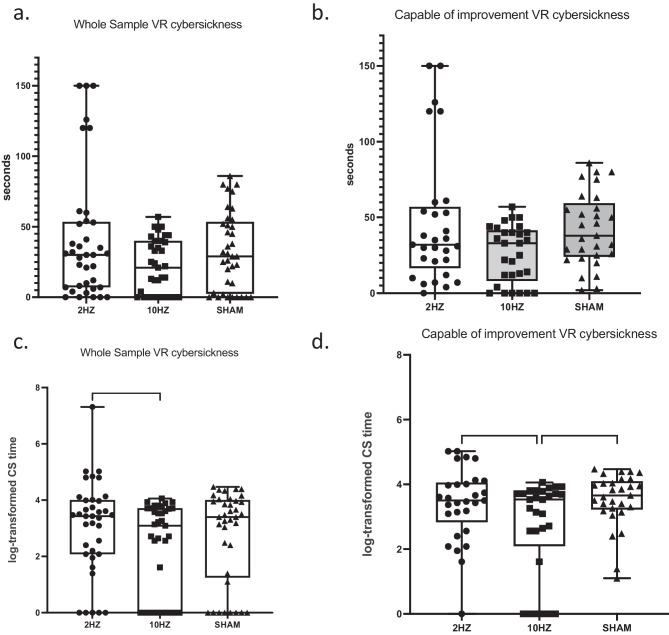
**a** Box plots show the raw differences between the mean length of the cybersickness symptoms that all participants reported during 2 Hz, 10 Hz and sham conditions. **b** Same organisation as **a**, but data refers to subjects capable of improvement  only. **c** Box plots show log-transformed values of CS experienced during 2 Hz, 10 Hz and sham conditions in the whole sample (* = *p* < .05). **d** Same organisation as **c**, but data refers to susceptible responders only (* = *p* < .05;*** = *p* < .001). In each panel, the graph components are as follows: line represents the median; boxers represent interquartile range (IQR); the box encloses not only the median but also the mean 50% of the data. Whiskers represent expected variation in the data and extend 1.5 times from the IQR from the top and bottom of the box

Thus, considering only those subjects who had a chance to improve their CS nausea, therefore called “capable of improvement”, the LMM assuming compound symmetry indicated that the null hypothesis of no difference between the three conditions could be rejected (F(2, 56) = 6.399, *p* = 0.003). The first planned comparison 10 Hz-tACS vs sham yielded a mean value of − 0.84 (95% CI: − 1.47, − 0.21; *p* = 0.005) and the second planned comparison 2 Hz-tACS vs sham in a mean value of − 0.11 (95% CI: − 0.74, + 0.52; *p* = 0.966). The comparison between the two real stimulations 10 Hz-tACS vs 2 Hz-tACS resulted in a mean value of − 0.73 (95% CI: − 1.36, − 0.10; *p* = 0.017). Thus, the primary outcome was met in this subsample because (1) the assumptions were close to the observations and (2) the observed effect size was slightly smaller than expected (Fig. 2).

As the observed differences could also be due to the treatment sequences, we first checked their balancing. The 6 stimulation sequences were randomly repeated: sham-2 Hz-10 Hz (7 repetitions), 10 Hz-sham-2 Hz (7 repetitions), 2 Hz-sham-10 Hz (5 repetitions), 2 Hz-10 Hz-sham (8 repetitions), 10 Hz-2 Hz-sham (5 repetitions) and sham-10 Hz-2 Hz (6 repetitions). Thus, the sham condition was presented 2 times as the first condition, 3 times as the second condition and 3 times as the third condition.

As a sensitivity analysis, we added order as a between-subjects factor and tested it as a main and interactive term with stimulation type. Neither the main effect nor the interaction was significant (F(5,23) = 1.242; *p* = 0.332 and F(10,46) = 1.270; *p* = 0.285, respectively (see Fig. S1, Supplemental Material).

On a descriptive level, 22 out of the 33 “capable of improvement” subjects (67%) improved their nausea during 10 Hz-tACS versus sham, whereas 18 out of the 41 subjects (44%) worsened their nausea during the rollercoaster ride during 2 Hz-tACS versus sham.

### Recovery Time and GSR

#### Recovery Time

The linear mixed model on recovery time on the whole sample was not significant (F(2,72) = 0.223; *p* = 0.80), suggesting that none of the stimulation conditions affected this variable.

The LLM on recovery time on “capable of improvement” subjects was again not significant (F(2,58) = 2.727; *p* = 0.073), suggesting that none of the stimulation conditions affected this variable.

Descriptively, a single subject (who had experienced nausea with subsequent vomiting during the sham condition and no discomfort during 10 Hz tACS) reported a long recovery time (112″) after the sham condition and immediate recovery (0″) for both 10-Hz and 2-Hz tACS conditions.

#### GSR

**Fig. 3:**
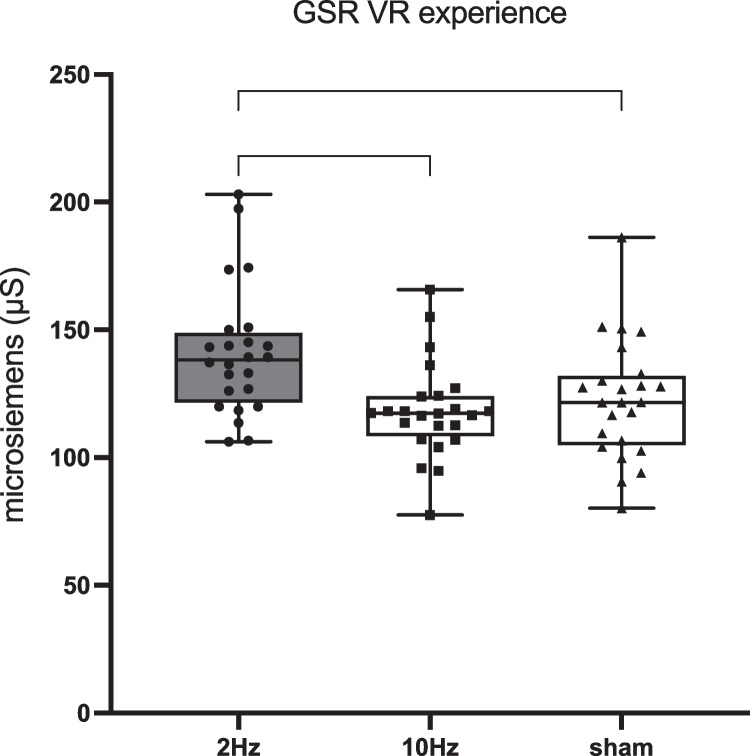
Box plots show variations of GSR activity recorded in 25 participants during the virtual reality experience throughout 2 Hz, 10 Hz and sham conditions (** = *p* < .01)

The LLM showed a significant effect of the applied stimulation frequency on GSR in the whole sample (F(2,46) = 9.262, *p* < 0.001; Fig. 3). Multiple comparisons showed a significant Bonferroni-corrected decrease in GSR during 10 Hz-tACS compared to the 2 Hz-tACS condition (*p* = 0.001) and a significant increase during 2 Hz-tACS compared to the sham condition (*p* = 0.005), but no significant difference between 10 Hz-tACS and sham.

### Correlations and Regressions

To test which factor between MSSQ scores and the level of nausea experienced during sham better predicted the reduction in nausea during 10 Hz-tACS, correlations analyses were performed using Spearman’s rho test.

In the whole sample of subjects, the difference in nausea between sham and 10 Hz-tACS conditions was significantly correlated with the nausea experienced during sham (*ρ* = 0.58; *p* < 0.001), but not with the MSSQ scores (*ρ* = 0.045; *p* = 0.79). Similarly, in “capable of improvement” sample, the difference in nausea between sham and 10 Hz-tACS conditions was almost significantly correlated with the nausea experienced during sham (*ρ* = 0.354; *p* = 0.060), but not with MSSQ scores (*ρ* = 0.102; *p* = 0.67) However, a relevant part of the correlations between the sham-10 Hz-tACS differences and nausea experienced during sham was due to the regression to the mean effect. When this effect was removed [18], no correlation was found (*ρ* = 0.28; *p* = 0.084) (see Fig. S2, Supplemental Material).

#### Side-Effect Questionnaire

The full statistical analysis of subjective side effects can be found in the Supplemental Materials. In general, subjective discomfort was significantly higher during 2 Hz-tACS than during 10 Hz and sham tACS. Slight headache, itching and tingling were similar for 2 Hz-tACS and 10 Hz-tACS, in both cases significantly higher than during sham stimulation (Fig. S3, Supplemental Material). Phosphenes were never reported, probably because masked by the VR immersion setting.

## Discussion

The primary aim of the current study was to verify the feasibility and efficacy of a noninvasive neuromodulatory technique as tACS of the vestibular cortex to reduce CS during VR experience, a very common users’ constellation of vestibular symptoms that are accompanied by disabling neurovegetative dysfunctions, in a real-life ecological setting.

The results show that 10 Hz-tACS, if biophysically modelled to adequately target the vestibular cortex bilaterally, could represent a new strategy to reduce CS during VR performance in the majority of users (indeed, 67% of subjects improved their level of nausea during the ride with 10 Hz-tACS), regardless of the order in which subjects received the stimulation. The improvement in nausea is partly supported by the GSR results, which showed a divergent effect between 2-Hz tACS (increase) and 10-Hz tACS (decrease) on the autonomic system function, as expected due to the strong physiological links between vestibular and neurovegetative activity. However, 10-Hz tACS did not differ from sham, suggesting a possible dissociation between the improvement of nausea and the decrease in sympathetic activity. Future studies should consider investigating of other neurovegetative parameters such as pupillometry and heart rate variability to obtain more precise information on both sympathetic and parasympathetic activity. In order to better disentangle whether GSR activity is influenced by tACS, VR effects or a combination of both, additional control conditions should include measurements of GSR activity during VR immersion (without tACS) as well as tACS alone (without VR immersion) at different frequencies.

The behavioural results cannot be explained by different side effects induced by the type of stimulation, as headache, itching and tingling scores were similar during stimulation at 2 Hz and 1 Hz. Only the discomfort score was marginally higher (see suppl. Mat., Fig. S3 and relative statistics) during 2 Hz-tACS than during 10 Hz and sham tACS: this might represent a confound in the interpretation of GSR increase during 2 Hz-tACS, as the general term discomfort may include both the induced sickness and the effects of the stimulation. It is worth noting that phosphenes, the most common side effect of 10 Hz-tACS [34], were not reported with the stimulation received during VR immersion, probably because the subjective report was masked by the peculiar luminance conditions of the VR environment.

As there was no correlation between individual susceptibility to CS, as measured by the MSSQ-short questionnaire [11, 26], and response to the stimulation, it can be postulated that tACS may be worth trying in all subjects experiencing nausea when undergoing to VR immersion. That the subjective questionnaire was not predictive of the subsequent response to 10 Hz-tACS may be due to the fact that the MSSQ is not specific for susceptibility CS but rather to kinetosis in general [35]. The fact that 2 Hz-tACS did not significantly worsen subjective nausea as did in normal subjects during posturographic testing [36] (although nausea increased in 44% of subjects) may be explained by the fact that the level of nausea during the ride was already quite high, making it difficult to be further significantly worsened by the stimulation.

However, as 10 Hz-tACS did not shorten the recovery time from the residual CS nausea, it should be considered only as an online CS countermeasure, at least when applied with the parameters of the current study. Further research is needed to verify whether prolonged tACS applications or repeated sessions of tACS during VR exposure (i.e. a kind of desensitisation approach) could lead to longer lasting improvements. It remains that other currently available strategies to reduce CS, such as self-controlled breathing [37], music [38], the use of references such as grid patterns or the nose [39] and artificial intelligence-enhanced six-degrees-of-freedom motion VR devices [39], are basically of little help in reducing CS nausea. Therefore, the 10 Hz-tACS applied during VR immersion may play an important role in reducing vestibular symptoms during VR immersion. Future studies should aim to verify whether alpha-tACS tuned to individual alpha activity or even higher frequencies of stimulation could have additive effects in reducing CS.

Beyond the training session, we did not use a baseline condition (i.e. without any tACS stimulation) to measure CS nor to test whether tACS alone could induce nausea, as this would have excessively prolonged the experimental time and has already been tested in a previous paper [13]. The use of sham as a reference may have contributed to the lack of power of post hoc comparisons on the whole sample of subjects. However, the order of the sham condition was counterbalanced and the order effect of the treatment sequences was null, suggesting the absence of carry-over effects of any type of tACS applied. This is also supported by the lack of improvement in recovery times.

NiBS techniques are increasingly being used as therapeutic procedures as an alternative or complementary to traditional pharmacological therapies for many neurological and psychiatric disorders, capitalising on the long lasting, predictable and safe [34, 40] after-effects of the stimulation interventions [41]. Among these, only a handful of previous pilot studies have used NiBS techniques to reduce vestibular symptoms of motion sickness or kinetosis: repetitive transcranial magnetic stimulation (rTMS) of the prefrontal cortex in cases of *mal de debarquement syndrome* [17, 42] and anodal transcranial direct current stimulation (tDCS) of the right temporoparietal junction applied before a VR rollercoaster game [43]: in this study, anodal tDCS improved the oculomotor but not the nausea sub-score of the Simulator Sickness Questionnaire after the VR experience, but no data are reported on online changes in nausea or other physiological assessment parameters, nor it is known whether online tDCS could have improved CS (or not).

The rationale for using rTMS or tDCS to reduce vestibular symptoms is thought to be based on the induction of predictable changes in excitability [albeit via different mechanisms of stimulation-neural interaction of the two techniques [44] in the targeted cortical regions [42, 43]. In the current study, tACS was preferred due to its unique ability to interact with endogenous oscillatory activity [40, 44, 45]. Typically, tACS is used because it is thought to entrain local oscillations in a frequency-specific manner [44], as recently shown in the vestibular domain where tACS at 1 Hz or 2 Hz induced motion sickness and postural sway in healthy subjects [16]. Here, prompted by the observation that 10 Hz-tACS reduced chronic symptoms in a patient with no peripheral vestibular function [16] and that improved persistent oscillatory vertigo following prolonged sea or air travel, even when not precisely targeted at the vestibular cortex [17], we reasoned that this kind of stimulation might be useful in reducing CS via at least two non-mutually exclusive mechanisms: first, that 10 Hz-tACS could reduce local slow wave activity that sustains symptoms [14] via phase interference [46], a common biophysical mechanism [47] useful, for example, in reducing pathological oscillatory brain activity that drives tremor [48]; second, as temporal-parietal alpha (i.e. 10 Hz) suppression appears to be a hallmark of the physiological activation of the multisensory vestibular cortex [18], the entrainment induced by 10 Hz-tACS on residual endogenous alpha oscillations (i.e. the opposite of the alpha suppression) in this range may disrupt the functioning of the vestibular regions and thereby also reduce symptoms. Direct answers to these hypotheses will emerge from EEG recordings performed immediately after the tACS application during VR immersion, a question we are investigating on EEG recordings collected in the context of the current experiment.

Clearly, potential applications of the current findings are relevant for several scenarios in which either cyber or motion sickness, which largely share common neural pathophysiological mechanisms [12], and/or related disturbances may occur. Beyond the alleviation of CS during gaming [49], we see of particular and immediate interest the possibility of facilitating VR training for surgeons [5], as well as for military scenarios, where soldiers play VR as avatars or as first person [6–8], or even more for disturbances during space missions performed in the absence of gravity [50, 51], since tACS devices are light and easily portable during space missions. Obviously, terrestrial clinical applications could also benefit from tACS during VR, for example, during prolonged psychotherapeutic sessions to treat symptoms of posttraumatic stress disorder through a systemic desensitisation process [4]. However, the most intriguing and potentially disruptive application of concurrent tACS to reduce CS during head-mounted VR devices may be the metaverse, considering its many possible exploitations for educational, relational and social purposes [52, 53]. Finally, tACS treatment could play an important role in self-driving vehicles where people may experience a significantly increased risk of motion sickness [54].

## Conclusions

Despite some of the current limitations discussed above, we have shown that 10 Hz-tACS applied to the vestibular cortex can reduce those CS symptoms that usually discourage VR use, as well as improve performance in regular VR users by reducing sub-threshold CS symptoms. The results may have relevant clinical implications, as they may be applicated to other vestibular dysfunctions beyond CS.

### Supplementary Information

Below is the link to the electronic supplementary material.

Supplementary file1 (DOCX 232 KB)

## Data Availability

The corresponding author has full access to all data and material and can provide availability if needed.
