# Supplementary material for: Frequency-Dependent Reduction of Cybersickness in Virtual Reality by Transcranial Oscillatory Stimulation of the Vestibular Cortex
Source: Neurotherapeutics. 2023 Sep 18;20(6):1796–807. doi: 10.1007/s13311-023-01437-6 (PMC10684476; doi:10.1007/s13311-023-01437-6)
Supplement: Supplementary file 1 — Supplementary file1 (DOCX 232 KB) [file 13311_2023_1437_MOESM1_ESM.docx]

Supplemental material for:

**Frequency-dependent reduction of cyber-sickness**

**in Virtual Reality by transcranial oscillatory stimulation**

**of the vestibular cortex**

Alberto Benelli ^1^, Francesco Neri ^1,2^, Alessandra Cinti ^1^, Patrizio Pasqualetti ^3^, Sara M. Romanella ^1,6^, Alessandro Giannotta ^1^, David De Monte ^1^, Marco Mandalà ^2, 4^, Carmelo Smeralda ^1^, Domenico Prattichizzo *^2,5^*, Emiliano Santarnecchi ^6,*^, Simone Rossi ^1,2,*^

* = these two authors contributed equally

*^1^ Siena Brain Investigation & Neuromodulation Lab (Si-BIN Lab), Unit of Neurology and Clinical Neurophysiology, Department of Medicine, Surgery and Neuroscience, University of Siena, Italy*

*^2^ Oto-Neuro-Tech Conjoined Lab, Policlinico Le Scotte, University of Siena, Italy*

*^3^ Health Statistics, University La Sapienza, Roma, Italy*

*^4^ Otolaryngology, Department of Medicine, Surgery and Neuroscience, University of Siena, Italy*

*^5^ Siena Robotics and Systems (SiRS) Lab, Department of Information Engineering and Mathematics, University of Siena, Siena, Italy*

*^6^ Precision Neuroscience & Neuromodulation Program, Gordon Center for Medical Imaging, Massachusetts General Hospital, Harvard Medical School, Boston, MA, USA*

**Running title**: Oscillatory neuromodulation for cyber-sickness

**Financial disclosures** All authors report no conﬂit of interest.

**Corresponding Authors:**

Emiliano Santarnecchi

Precision Neuroscience and Neuromodulation Program,

Gordon Center for Medical Imaging, Department of Radiology,

Massachusetts General Hospital,

Harvard Medical School, Boston, MA, USA

Simone Rossi

Siena Brain Investigation & Neuromodulation Lab (Si-BIN Lab), Unit of Neurology and Clinical Neurophysiology, Department of Medicine, Surgery and Neuroscience, University of Siena, Italy

Tel: 0577 233 321

Email: [simone.rossi@unisi.it](mailto:simone.rossi@unisi.it)

Website: [www.sibinlab.it](http://www.sibinlab.it)

*The research has received partial funding from the European Commission H2020 Framework Programme under Grant No. 101017727 of the project “EXPERIENCE”*

*Authors are grateful Prof. Francisco Javier Cudeiro Mazaira for a critical revision of the manuscript.*

**Results**

The supplementary Figure 1 summarizes the level of CS experienced by subjects along the six orders of sequence of treatments.

The supplementary Figure 2 shows that the correlation between baseline CS sham (x-axis) decreased after the adjustment for regression-to-the-mean effect (from r=0.58, p<0.001 to r=0.28, p=0.084).


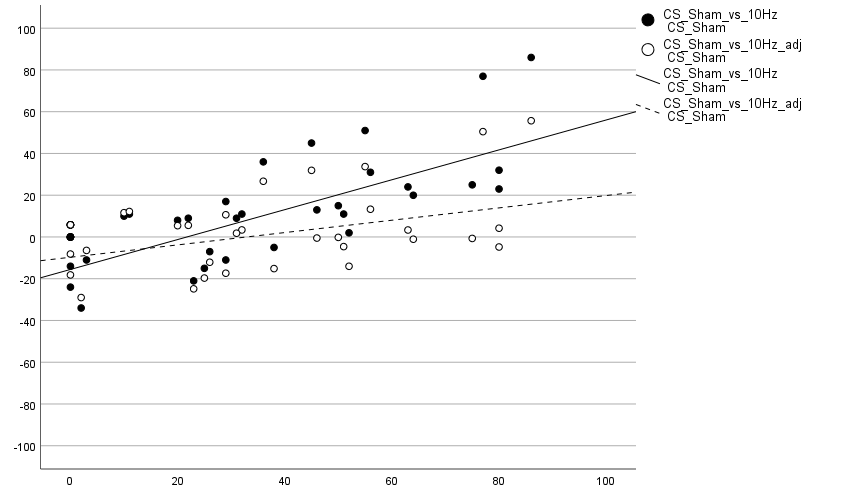


***Side effects questionnaire***

The questionnaire has a range of scores from 0 to 4. The Linear Mixed Model showed a significant effect of the applied frequency of stimulation on the headache score (F(1.793,64.56)=17.87, p < .05). Multiple comparisons showed a significant increase during 10 Hz-tACS versus Sham condition (p= .0001), a significant increase during 2 Hz-tACS versus Sham condition (p= .0001), but no significant difference between 10 Hz-tACS and 2Hz.

The Linear Mixed Model showed a significant effect of the applied frequency of stimulation on the itching score (F(1.793,64.56)=17.87, p < .05). Multiple comparisons showed a significant increase during 10 Hz-tACS versus Sham condition (p= .0001), a significant increase during 2 Hz-tACS versus Sham condition (p= .0001), but no significant difference between 10 Hz-tACS and 2Hz. Multiple comparisons showed a significant increase during 10 Hz-tACS versus Sham condition (p= .0001), a significant increase during 2 Hz-tACS versus Sham condition (p= .0001), but no significant difference between 10 Hz-tACS and 2Hz.

The Linear Mixed Model showed a significant effect of the applied frequency of stimulation on the tingling score (F(1.803,97.38)=13.22, p < .05). Multiple comparisons showed a significant increase during 10 Hz-tACS versus Sham condition (p= .0001), a significant increase during 2 Hz-tACS versus Sham condition (p= .0003), but no significant difference between 10 Hz-tACS and 2Hz.

The Linear Mixed Model showed a significant effect of the applied frequency of stimulation on the discomfort score (F(1.694,91.49)=14.73, p < .05). Multiple comparisons showed a not significant increase during 10 Hz-tACS versus Sham condition, a significant increase during 2 Hz-tACS versus Sham condition (p= .0001), a significant increase at 2Hz compared to 10Hz-tACS (p= .0255). Fig.4. Participants didn’t report phosphenes (probably masked by VR). It is not easy to distinguish in the subjective reports how much what they reported was more attributable to the effect of stimulation alone, VR, oculus weight or the combination of these factors.

**Legends to supplementary figures**

**Figure S1, Supplemental Material**. The Figure shows the level of CS experienced by subjects along the six orders of sequence of treatments. The Sham-2Hz-10Hz sequence occurred 7 times; the 10Hz-Sham-2Hz occurred 7 times; the 2Hz-Sham-10Hz sequence occurred 5 times; the 2Hz-10Hz-Sham sequence occurred 8 times; the 10Hz-2Hz-Sham sequence occurred 5 times; the Sham-10Hz-2Hz occurred 6 times. Reduction of CS during 10 Hz-tACS occurred in 5/6 sequences. The occurrence of Sham condition was balanced, as it was presented 2 times as the first condition, 3 times as the second condition, and 3 times as the third condition.

**Figure S2, Supplemental Material.** Decrease of the correlation between baseline CS sham (x-axis) after the adjustment for regression-to-the-mean effect

**Figure S3, Supplemental material**. Graphs show the values of side effects due to the 3 different conditions in the subcategories of headache, itching, discomfort and tingling (*=p<.05; ***=p<.001; ****=p<.0001). Graphs made with GraphPad Prism.
